# Nucleation status of Day 2 pre-implantation embryos, acquired by time-lapse imaging during IVF, is associated with live birth

**DOI:** 10.1371/journal.pone.0274502

**Published:** 2022-09-22

**Authors:** Shabana Sayed, Marte Myhre Reigstad, Bjørn Molt Petersen, Arne Schwennicke, Jon Wegner Hausken, Ritsa Storeng

**Affiliations:** 1 Klinikk Hausken, IVF and Gynecology, Haugesund, Norway; 2 Norwegian Research Centre on Women’s Health, Oslo University Hospital, Oslo, Norway; 3 BMP Analytics, Viby J, Denmark; Public Library of Science, UNITED KINGDOM

## Abstract

The primary purpose of this time-lapse data analysis was to identify the association between the nucleation status of a Day 2 preimplantation embryo and live births following *in vitro fertilization* (IVF). The retrospective data analysis was based on 2769 transferred embryos from 1966 treatment cycles and utilised only Known Implantation Data (KID) for live births. Nucleation errors (NE) such as micronucleation, binucleation, multinucleation and minor error groups, were annotated in the time-lapse images which were taken every 15 minutes for a minimum of 44 hours post insemination. Further, factors that may impact NE and the relationship of early morphological attributes and morphokinetic variables with NE occurrence were explored. The frequency of NE among the transferred embryos was 23.8%. The reversibility of NE evidenced by their presence at the two-cell stage, but absence at the four-cell stage was 89.6%. Embryos exhibiting nucleation errors at the two-cell stage had significantly lower live birth rates compared to embryos with no nucleation errors, constituting a significant predictor. A Generalized Additive Mixed Model was used to control for confounders and for controlling clustering effects from dual embryo transfers. Increased incidences of NE were observed with increasing age, with delayed occurrence of cell divisions and in oocytes inseminated with surgically retrieved spermatozoa. NE assessment and their impact on live birth provides valuable markers for early preimplantation embryo selection. In addition, the high incidence of reversibility of NE and their possible impact on live birth suggest that incorporating two-cell nuclear status annotations in embryo selection, alongside morphology and morphokinetics, is of value.

## Introduction

The ideal clinical outcome following *In vitro* fertilisation (IVF) treatment is the ´birth of a healthy singleton child at term´ [1–4]. Even though single embryo transfer has been widely adopted, high risk multiple pregnancies remain a problem. Reduction of multiple gestations is achieved by limiting the number of embryos transferred to one, whenever possible. To achieve this without compromising success rates, selection of embryos with the highest implantation and live birth potential is of key importance [5–7].

Embryo selection based on morphology has been a long-standing gold standard in IVF treatment regimens [8, 9]. With the advent of time-lapse imaging (TLI) technology, additional non-invasive biomarkers, morphologic and morphokinetic, have been available to embryologists, aiding in embryo selection and / or deselection for transfer and cryopreservation [10–13].

TLI provides the possibility to continuously observe the nucleation status of an embryo, as opposed to intermittent observations at fixed time intervals. This continuous monitoring may provide additional information to embryologists, facilitating embryo selection [14]. Hnida et al. [15] for instance, observed that only 26% of nucleation errors (NE) were detected when using traditional scoring methods, relative to computer-controlled non-invasive multilevel analysis. The presence and proportion of visible nuclei in blastomeres have been indicated as a prime selection marker at four-cell preimplantation embryo and have been associated with significantly higher implantation rate [16, 17], pregnancy [6, 18–21] and live births [13].

The presence of multinucleated blastomeres (MNB) in early preimplantation embryos has been used as a deselection parameter during embryo selection for transfer and cryopreservation [22–25]. The reported frequency of multinucleation ranged from 17% to 69% [14, 26–29] and multinucleation has been used as a deselection biomarker for embryo transfer and cryopreservation [20–26].

NE in embryos have been shown to indicate impaired embryonic development, low rates of implantation and high miscarriage rates [29]. Furthermore, the presence of one visible nucleus in each of the blastomeres at the four-cell stage have been associated with significantly higher implantation [16, 17, 20, 29] and live birth rates (LBR) [13]. Few studies have assessed the association of NE with live birth rate as the primary endpoint [13, 25, 30]. Even fewer studies have attempted to characterise the nucleation error patterns [14, 25] observed by TLI and their potential association with LBR.

The aim of this analysis is to retrospectively assess the occurrence of NE as well as nucleation error phenotypes (NEP) as observed by TLI and to examine their association with live birth following IVF treatments. The study further aims at investigating factors that may have an impact on NE occurrence by exploring the relationship of early embryo morphological attributes and morphokinetic variables with the occurrence of NE.

## Materials and methods

This retrospective observational study is based on patient data from Klinikk Hausken, Haugesund, Norway between May 2011 through August 2018. A total of 2769 transferred embryos derived from 1966 treatment cycles with complete live birth-known implantation data (LB-KID) information were included in the analyses. All embryos were cultured in the EmbryoScope® time-lapse imaging system (Vitrolife; Sweden) for a minimum of 44 hours from fertilisation check until embryo transfer (ET).

The outcome measure was LB per transfer, confirmed by a clinical delivery reported either by the patient to the clinic or retrieved from the Medical Birth Registry of Norway (MBRN).

### Ovarian stimulation, insemination, and embryo culture

Two controlled ovarian stimulation protocols were used, either agonist protocol with midluteal down-regulation using a gonadotropin releasing hormone (GnRH) agonist (Synarel®; Pfizer Limited, UK) or antagonist protocol (ganirelix injection; Orgalutran; Merck Sharp & Dohme B.V; Netherlands) as elaborated by Sayed et al. [31]. Oocyte retrieval was performed by transvaginal ultrasound-guided needle aspiration of the follicles, 36 hours after human chorionic gonadotropin (hCG) (Ovitrelle; Merck Serono) trigger. The origin of sperm samples varied from ejaculation to surgical sperm retrievals; percutaneous epididymal sperm aspiration (PESA), testicular sperm aspiration (TESA) or testicular sperm extraction (TESE). The type of insemination, either conventional in vitro fertilisation (IVF) or Intracytoplasmic sperm injection (ICSI), depended on the sperm quality as per WHO standards (2010) [32]. ICSI was the choice of insemination for severe oligozoospermia (sperm count < 10 million /ml), asthenozoospermia (< 32% progressive motile sperms) and severe teratozoospermia (strict normal sperm morphology ≤ 4%) based on previously published studies [33–35].

EmbryoSlide^TM^ (Vitrolife, Sweden) culture dish preparation and overnight equilibration were performed at 37° C with 6% CO_2_ and 5% O_2_ in humidity set incubators following Standard operating procedure (SOP) as elaborated in Sayed et al. [31]. The presence of two pronuclei and two polar bodies, 16–18 hours after insemination indicated normal fertilisation and these normally fertilised zygotes were transferred to the pre-equilibrated EmbryoSlides^TM^ and subsequently cultured in the EmbryoScope^TM.^ until ET.

### Time-lapse imaging system and patient data

Time-lapse videos were used to annotate cell stages, degree of fragmentation, symmetry of cells as well as cell cleavage patterns and nucleation status of the embryos. In addition, the timings of specific cell cycle events were expressed as hours post insemination (hpi) and zygotes were transferred to the EmbryoScope at 16–18 hpi. The Embryo Viewer software (Vitrolife, Denmark) and the treatment database IDEAS (Mellowood Medical, Canada) stored all patient and treatment information. Unique patient registration number and treatment cycle identification gave insight into patient characteristics, etiology and stimulation responses as further described in Sayed et al. [31].

### Embryo assessment

The Embryo Viewer image analysis software (Vitrolife; Denmark) was used by the embryologists to annotate the precise timings of cell cycle events as well as nucleation status. Annotations were performed sequentially following the laboratory Standard operating procedures to minimise inter and intra-observer variability [31]. The annotations were double- checked by two embryologists to confirm annotations as well as to avoid missing annotations.

### Morphokinetic annotations

The morphokinetic annotations were cell stage specific and were carried out according to Meseguer et al. [36]. The following were annotated: fading out of the two pronuclei (tPNf), exact timings of embryo cleavages, appearance of two-cells (t2), three-cells (t3) and four-cells (t4). Second cell cycle duration (cc2 = t3-t2) and the synchrony in divisions (s2 = t4-t3) were considered crucial for Day 2 annotations. The PN duration, VP was also calculated to denote the time between the fading of the two pronuclei and the appearance of a two-cell embryo (t2-tPNf). In comparison to ICSI embryos, there is an average 0.28 hours delay in tPNf for the IVF embryos. This average time lag between IVF and ICSI embryos is lower than reported lags in previously published studies [37, 38]. In this analysis there is distinction between IVF and ICSI embryos in the GAMM analysis, but no attempt was taken to normalize the timings between IVF and ICSI.

### Nucleation status

Nucleation status was annotated for Day 2 embryos. This included the number of nuclei in each blastomere during interphase and the number of blastomeres with at least one nucleus as described by Ciray et al. [39]. Embryos with one visible nucleus per blastomere were prioritised for embryo transfer [17]. At the two-cell stage, nucleation error was recorded as multinucleated blastomeres MNB ^2cell.^ These were further annotated as MNB ^2cellx1^ or MNB ^2-cellx2^, depending on the presence of the error in one or both the blastomeres of a two-cell embryo respectively. If nucleation error was evident at the four-cell stage, it was annotated as MNB ^4 cell^ and additionally, the type of NEP was also noted.

**Fig 1:**
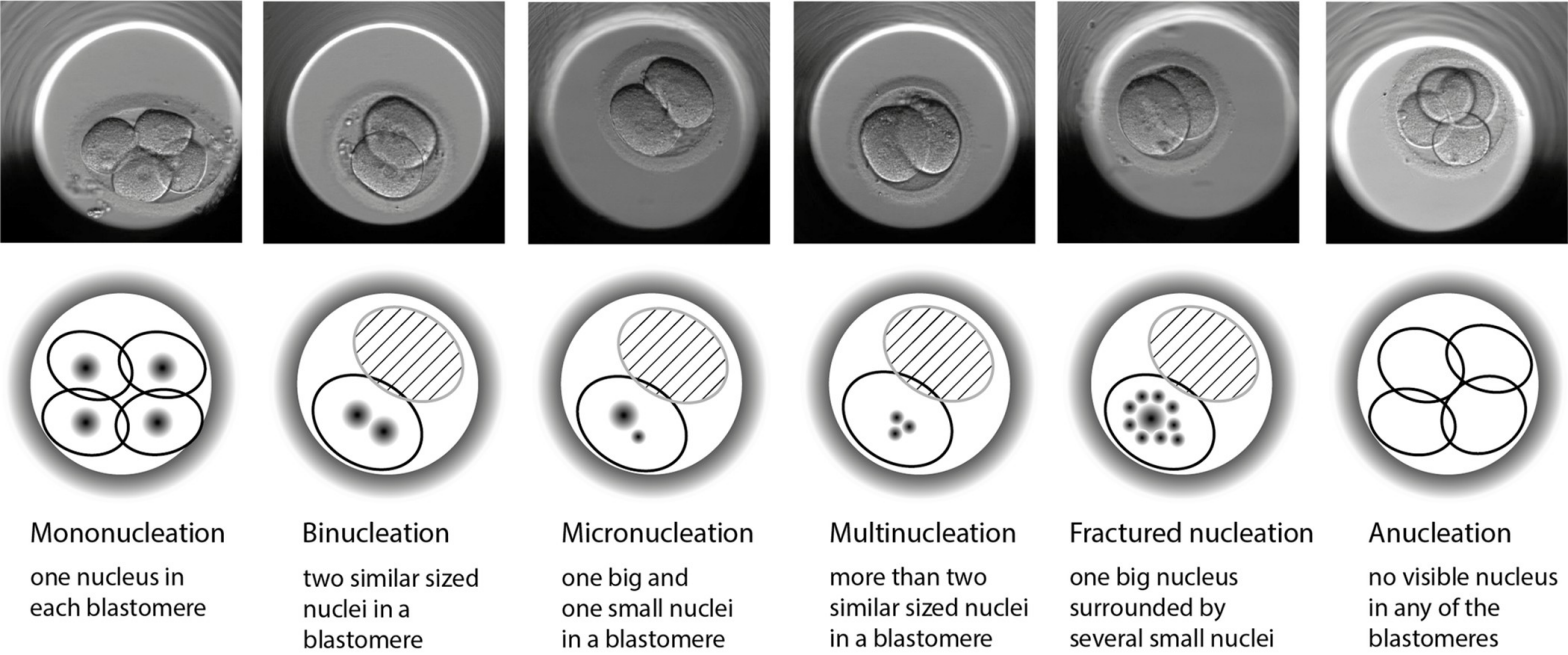
Illustration of the different nucleation error phenotypes (NEP) in early preimplantation embryos.

The NEPs were further annotated as binucleation (presence of two evenly sized nuclei per blastomere), micronucleation (one or two smaller nuclei surrounding a larger nucleus with nucleoli), multinucleation (more than two similar sized nuclei per blastomere), fractured nucleation (one or two larger nuclei surrounding several smaller nuclei) and anucleation (no visible nucleus) as illustrated in Fig 1.

Reversibility of nucleation errors, as described by Aguilar et al. [18], was calculated as the proportion of embryos with NE at the two-cell stage, but not displaying NE at the four-cell stage.

### Embryo selection, transfer, and luteal support

Embryo transfers were predominantly performed on Day 2 (78%) with the remaining transfers performed either on Day 3 (20%) or Day 5 (2%) during the study period. Morphological grading of Day 2 embryos included assessment of the number and symmetry/equality (even or uneven) of blastomere size, degree of fragmentation and presence or absence of a visible nucleus in the blastomeres [40]. The degree of fragmentation was divided into 3 categories: mild (0–10% fragmentation), medium (10–20%) and severe (>20%). If there were more than one recorded fragmentation, the highest value was used for the analysis.

Embryo selection on Day 2 was primarily based on morphology, namely cell numbers, symmetry of the cells, percentage of fragmentation, nucleation status as well as absence of any cleavage anomalies during early pre-implantation development. De-selection criteria for embryo transfer were based on cleavage anomalies and nucleation errors. Direct cleavages connected with reduced implantation potential of an embryo [41, 42] were not preferred for transfer.

In the absence of good quality embryos available for transfer, embryos with NE were considered. Morphologically good quality embryos exhibiting MNB ^2cellx1^ and no visible NE at the four-cell stage were preferred for embryo transfer when no other embryo was available. Morphologically good quality embryos exhibiting MNB ^2cellx2^ having no visible NE in the four-cell stage were transferred after consultation with the doctor and the couple undergoing treatment. If the only available embryo for transfer exhibited reversibility of nucleation error, the couple was informed, and consent taken before proceeding with embryo transfer. The patient’s previous infertility history, female age and embryo quality decided the number of embryos selected for transfer.

Pre-equilibrated Embryo Glue (Vitrolife; Sweden) was used as the transfer media and transabdominal ultrasound guided embryo transfer was performed. Luteal support using intravaginal progesterone capsules (Lutinus; Ferring; 100 mg/td) continued up to at least the day of positive or negative pregnancy test, 14 days after oocyte collection.

### KID analysis and outcome measures

KID values are based on traceability of all embryos [43, 44]. Partial live birth implantations resulting from transfer of multiple embryos makes it impossible to trace which of the transferred embryo had implanted. Hence, these partial implantations are not included in KID analyses [43].

LB rate, the ratio between the number of live births and the number of embryos transferred, was the endpoint of the study. For the subset of KID embryos, the LB rate was calculated as 100% x KID positive/ (KID positive + KID negative).

### Statistical analyses

Fisher’s exact test was used for univariate comparisons of categorical data between groups, including the evaluation of LB for their association to nucleation errors. Results were considered significant at *P* < 0.05. Odds ratios (OR) were calculated from contingency tables. For describing the association between nucleation errors and kinetic TLI variables, timings were converted from continuous variables to categorical variables by grouping them into quartiles. The quartile intervals are given in hours and denoted for each variable.

Because patient age and t2 had clear non-linear effects on the probability of birth and the probability of NE and further given that we can have more than one observation from a given patient, a Generalized Additive Mixed Model (GAMM) was applied. The model allows for nonlinear functional shapes between variables and the response, and further allows for minimising the potential clustering effect from dual embryo transfers by using a patient random effect.

The model responses are expressed as OR, the 95% confidential interval (CI) and the *P* value. To assess the combined effect of central variables and their possible confounding effects, two separate GAMM models were used, one for predicting the probability of LB and another for predicting the probability of NE occurrence. The utilised predictors (covariates) for the GAMM models were patient age, Body Mass Index (BMI), nucleation error (only for the first model), t2 (h), transfer at day 2, short second cell cycle (t3—t2 < 9.33 h), long second cell cycle (t3—t2 > 12.65 h), fragmentation (%), uneven blastomeres at the 2-cell stage, fertilisation using ICSI or surgical sperm retrieval (PESA, TESA, TESE). The durations for the short and long cycles are taken from VerMilyea et al. (2014) [45].

The ORs regarding live birth and nucleation error occurrence and the statistical significances were compared for univariate results versus the multivariate regressions. A direct comparison is only possible for categorical variables.

All statistical analyses were performed using the R statistical software package (R Foundation for Statistical Computing; Vienna, Austria).

### Treatment and patient characteristics

The present analysis includes 2769 transferred embryos derived from 1966 Assisted Reproductive Technology (ART) treatment cycles, with an average of 1.41 embryos transferred per cycle. Of the treatments having complete KID information, 1163 constituted single embryo transfers (SET) and the remaining 803 constituted double embryo transfers (DET).

The mean age of the patients was 35.5 years (SD 5.3), and mean BMI was 25.0 (SD 5.1) kg m^- 2^. There were 277 patients that had one previous treatment, 98 that had two previous treatments and 48 that had three previous treatments or more. Only previous treatment within the Hausken clinic chain were recorded.

The main treatment diagnoses are Male factor (39%), Endometriosis (18%), PCO (21%), Tubal factor (7%), Anovulation (6%) and other diagnoses (9%). In 18% of the treatments there was no diagnosis. The median number of aspired oocytes are 9 (1–29). The types of fertilisation methods are Insemination (IVF) 51%, Injection (ICSI) 40%, PESA 3%, TESA 2% and TESE 4%.

Embryo transfers during the study period were predominantly performed on Day 2 (78% of embryos) due to clinician’s preferences as well as patient logistics.

### Ethical approval

The Regional Committee for Medical and Health Research Ethics (REC) (2017/1610) approved the study protocol for this data analysis of embryos. The informed consent for IVF treatment provided by the clinic at the start of treatment specifically did not include the consent for retrospective data analysis. Therefore, consent specific to the study was sent out to all couples who underwent treatment during the study period. According to the written reply, 13 couples did not wish to participate. This was documented in their clinic files, and these treatments were not used in the study. The procedure was approved by the Ethics committee. Further, all data pertaining to the study period were fully anonymized before accessing them for the study.

## Results

### Frequency of NE in Day 2 embryos

Among the 1966 ART treatment cycles included in the analysis, 574 (29.2%) had at least one embryo in their entire embryo cohort with NE. Of the 2769 transferred embryos, 656 exhibited NE. The incidence of NE among the transferred embryos was 23.8%. Among the 656 embryos exhibiting NE, 634 (96.6%) had NE at the two-cell stage and 91 (13.9%) had NE recorded at the four-cell stage. Among the latter, 25 embryos did not display visible NE at the two- cell stage, but only at the four-cell stage. Among the transferred embryos, the percentage exhibiting NE in the two-cell stage in one blastomere was 21.8% and 2.0% had NE in both blastomeres.

Of all different NEP types among the transferred embryos, 202 were binucleated embryos and this NEP type was the most common (30.7% of all NEP’s). The reversibility of NE evidenced by the presence of NE at the two-cell stage but absent from the four-cell stage was 89.6%.

### Multivariate odds ratio results

The multivariate odds ratios (ORs) are used to compare the odds of the occurrence of LB, respectively the odds for occurrence of NE, relative to exposure to the variables of interest. In the multivariate analysis predicting live birth occurrence, the following variables had a statistically significant association: patient age, t2, BMI, nucleation error, transfer at day 2, short second cell cycle, long second cell cycle and fragmentation level (Table 1).

**Table 1:** Multivariate GAMM analysis for live birth.

Variable	Odds ratio	95% CI		*P*
BMI	0.98	0.96	1.00	<0.05
Nucleation error	0.62	0.46	0.83	<0.002
Transfer at day 2	0.69	0.54	0.88	<0.003
Short second cell cycle (t3—t2 < 9.33 h)	0.50	0.29	0.88	<0.02
Long second cell cycle (t3—t2 > 12.65 h)	0.62	0.45	0.83	<0.004
Fragmentation (%)	0.99	0.98	1.00	<0.02
Uneven blastomeres at the 2-cell stage	0.76	0.49	1.19	0.24
ICSI	1.00	0.80	1.26	0.99
PESA, TESA, TESE	0.86	0.58	1.28	0.46

The curves do not have a fixed odds ratio. The curve for age has *P* < 0.0001 and t2 has *P* < 0.001.

In the multivariate analysis used to predict nucleation error occurrence, patient age, t2, transfer at day 2, uneven blastomeres at the 2-cell stage, the use of ICSI and the use of either PESA, TESA or TESA all were significantly associated with the occurrence of NE (Table 2).

**Table 2:** Multivariate GAMM analysis for nucleation error.

Variable	Odds ratio	95% CI		*P*
BMI	0.99	0.98	1.01	0.63
Transfer at day 2	0.74	0.59	0.91	0.006
t2 (h)	1.07	1.03	1.10	<0.0002
Short second cell cycle (t3—t2 < 9.33 h)	1.00	0.69	1.44	0.98
Long second cell cycle (t3—t2 > 12.65 h)	1.13	0.84	1.44	0.31
Fragmentation (%)	1.00	1.00	1.01	0.17
Uneven blastomeres at the 2-cell stage	1.58	1.17	2.14	0.003
ICSI	1.80	1.17	2.14	< 0.0001
PESA, TESA, TESE	2.41	1.74	3.5	< 0.0001

The curve for age (*P* < 0.002) does not have a fixed odds ratio.

Some of the ORs regarding live birth and nucleation error occurrence and the statistical significances were compared for univariate results versus the multivariate regressions.

### Association of NE and NEPs with live birth rates

**Fig 2:**
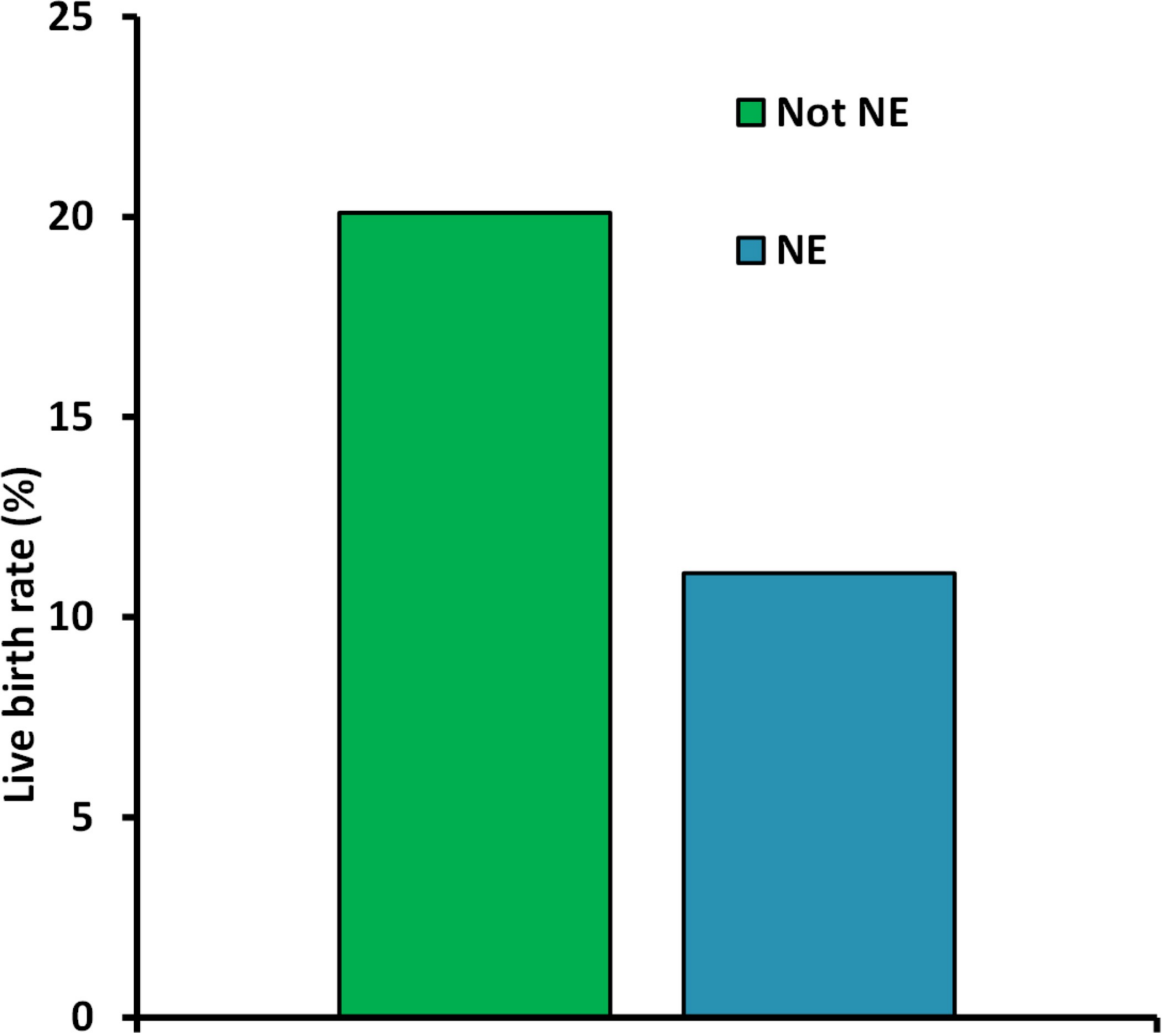
Live birth rate (LB) according to nuclear status of the transferred embryos. *P* < 0.001.

Embryos with NE were associated with lower live birth rates than those without any NE (11.1% vs. 20.1%; *P* < 0.001) (Fig 2).

On average, the transfer of an NE embryo had an OR regarding live birth of 0.50, *relative* to an embryo with no visible NE (univariate analysis).

In the multivariate analyses, the corresponding OR for live birth with NE occurrence was 0.62 (*P* < 0.002).

**Fig 3:**
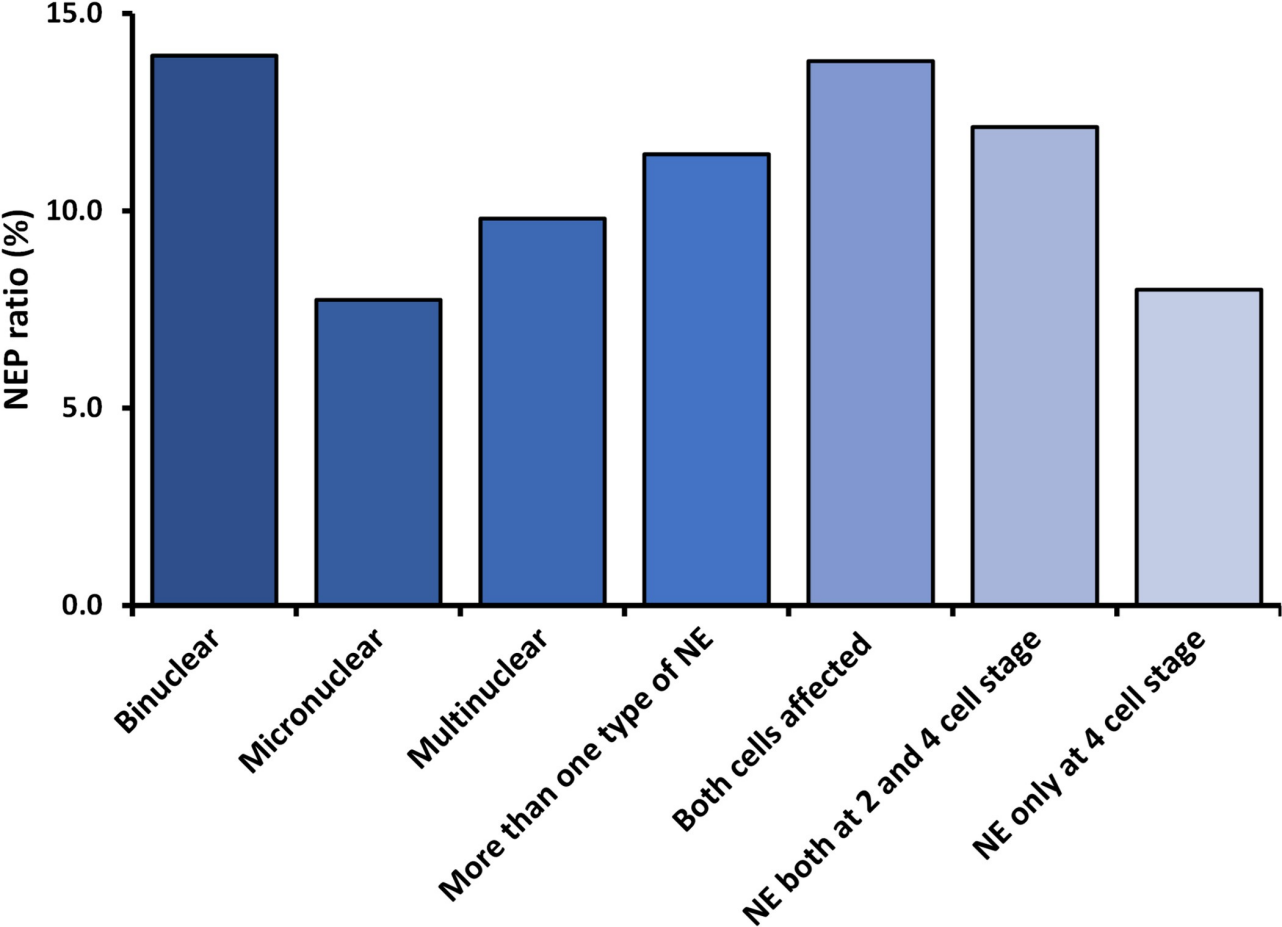
Nucleation error phenotypes (NEP) and effect on Live birth rate (LB-KID).

The association of various NEP types with LB-KID rates are shown in Fig 3.

Within the cohort of embryos with NE, there are no statistically significant differences in LB-KID rates between the different NEPs. For example, the number of embryos exhibiting NE in both blastomeres in a two-cell stage (n = 58) resulted in 13.8% live births (n = 8) which is comparable to the LB-KID rate of 11.1% for all NE occurrences.

### Morphokinetics and fragmentation

Table 3 shows early embryo morphokinetic variables grouped in quartiles and their relation to NE occurrence. Embryos occurring in the fourth quartile (Q4) have higher average incidence of NE for all the morphokinetic variables. For instance, embryos that cleaved to two-cells (t2) ≥ 29.42 hpi had a higher incidence of developing NE than those that had t2 ≤ 25.37 hpi (31.3% vs. 19.3%).

**Table 3:** Timing of the morphokinetic variables from 2769 transferred embryos grouped in quartiles Q1, Q2, Q3 and Q4 with nucleation error (NE) occurrence.

			Q1		Q2		Q3		Q4
Variable	No. of embryos	Limit (Hours)	NE rate (%)	Limit (Hours)	NE rate (%)	Limit (Hours)	NE rate (%)	Limit (Hours)	NE rate (%)
tPNf	1703	≤ 22.57	18.3**	22.58–24.46	21.7	24.47–26.53	21.7	≥26.54	29.2***
t2	2764	≤25.37	19.3***	25.38–27.20	22.2	27.21, 29.41	22.2	≥ 29.42	31.3***
VPN	1700	≤2.34	19.3	2.35–2.67	22.4	2.68–2.99	22.4	≥ 3.00	26.8*
t3	2542	≤36.08	19.2**	36.09–38.40	21.1	38.41–40.80	21.1	≥ 40.81	29.7***
cc2	2542	≤10.67	23.4	10.67–11.51	18.6**	11.51, 12.34	18.6**	≥ 12.34	27.7**
t4	2428	≤36.99	18.1**	37.00–39.33	20.4	39.34–41.65	20.4	≥ 41.66	30.5***
t4—t2	2426	≤11.34	20.1	11.35–12.30	20.4	12.31–13.17	20.4	≥ 13.18	28.6***
S2	2426	≤0.33	20.6	0.34–0.66	18.8**	0.67–1.16	18.8**	≥ 1.17	27.2**

* *P* < 0.05.

**P < 0.01.

***P < 0.001.

**Fig 4:**
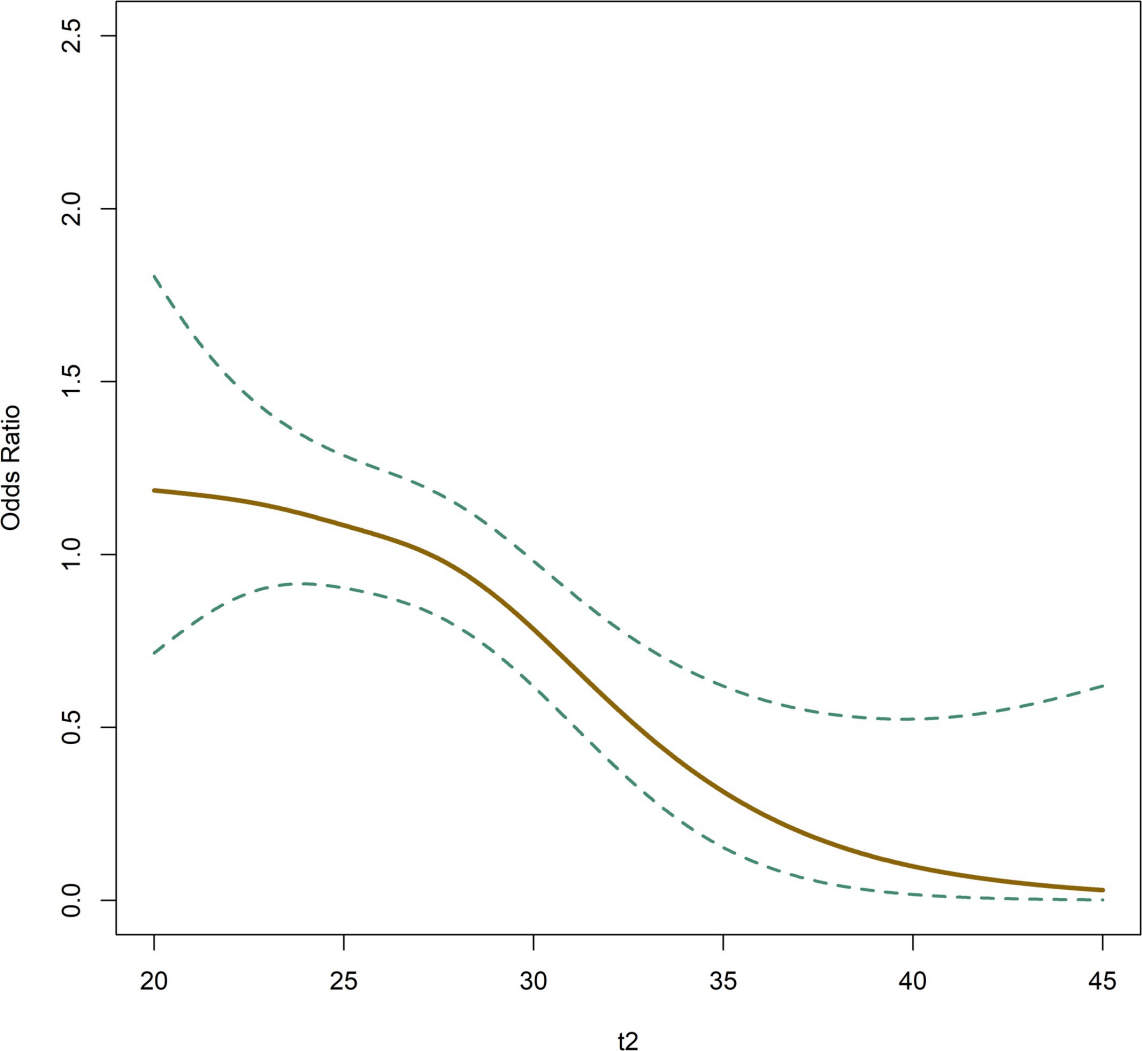
Odds ratio regarding live birth relative to t2 (first cell division, hours after insemination). Dashed lines show the 95% confidence interval (CI).

The multivariate analyses show a strong effect of late t2 for both LB (Fig 4; *P* < 0.001) and NE (OR = 1.07; *P* < 0.0002).

The duration of the second cell cycle, cc2, is a possible predictor for NE occurrence using cc2 timing thresholds derived from the EEVA model (second version; cc2<9.33h) [45]. When the duration of the second cell cycle was less than 9.33h, a pronounced reduction in LB-KID rate and an increase in NE occurrence was observed as shown in S1 Table.

According to the multivariate analysis, the occurrence of cc2<9.33h “short second cell cycle” (Table 1) versus a “normal” cycle yields an OR for LB of 0.50; (*P* < 0.02). A “long second cell cycle” (Table 1) is also associated with clearly reduced LB probability (OR = 0.62; *P* < 0.004).

A significant reduction in LB-KID rates and an increase in the incidence of NE occurrence were associated with increasing embryo fragmentation levels (Tables 1 and 2 and S2 Table).

### Patient and treatment cycle characteristics

There is a higher incidence of NE with increasing patient age in Table 2 (*P* < 0.002). Further, as a well-known association, Table 4 shows a distinct decline in live birth ratio with increasing age.

**Table 4:** Association between age, live birth, and nucleation error occurrence. Odds ratios are calculated on a univariate basis.

Age (y)	N	LB rate NE absent (%)	LB rate NE present (%)	NE (%)	Odds ratio NE absent	Odds ratio NE present
< 30	501	36.2	18.4	20.6	2.01	1.03
30–35	725	29.4	17.2	20.8	1.64	0.96
35–40	854	15.1	10.6	25.5	0.84	0.59
> 40	689	3.2	2.7	26.7	0.18	0.15

**Fig 5:**
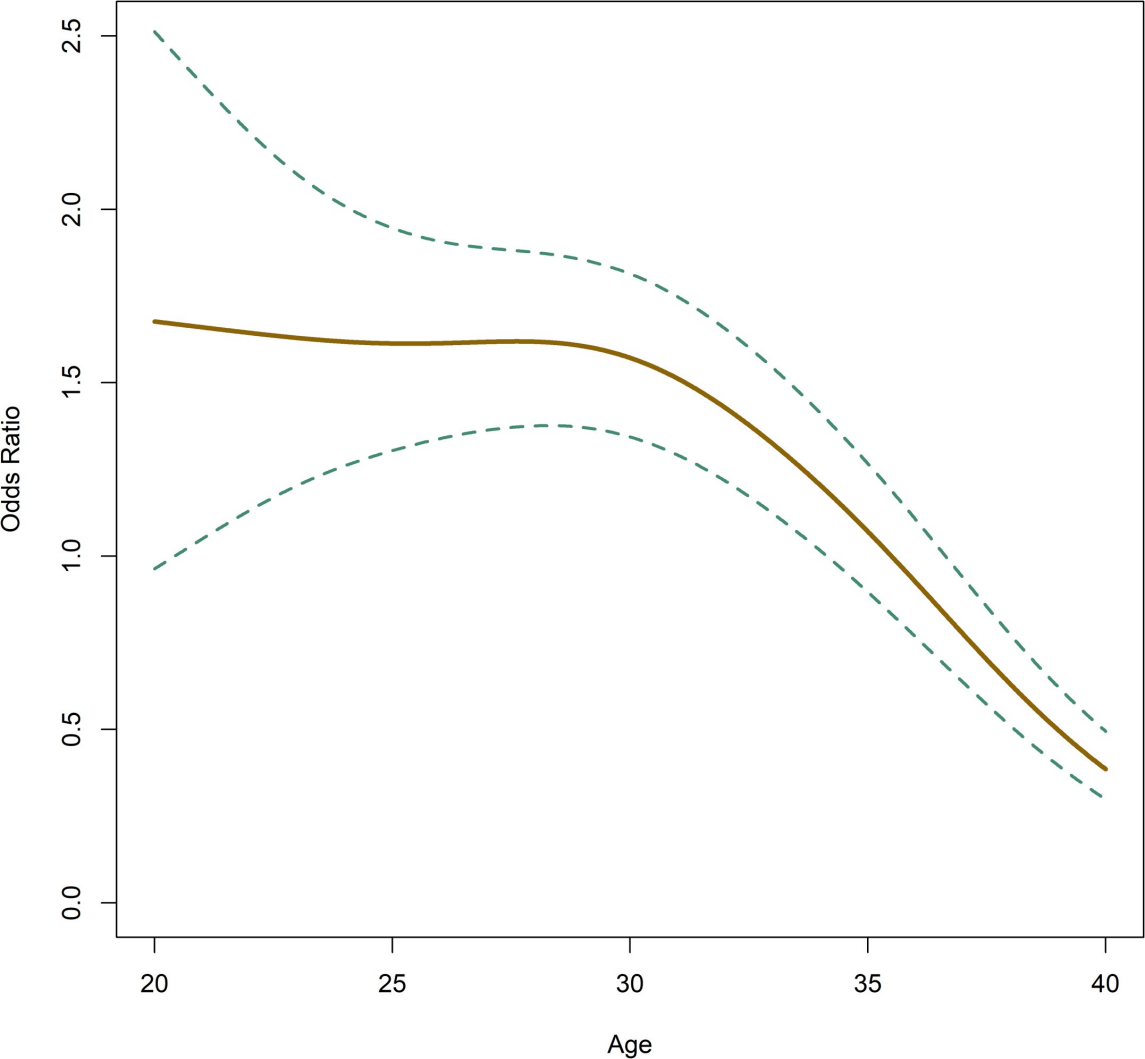
Odds ratio regarding live birth relative to patient age. Dashed lines show the 95% confidence intervals (CI).

**Fig 6:**
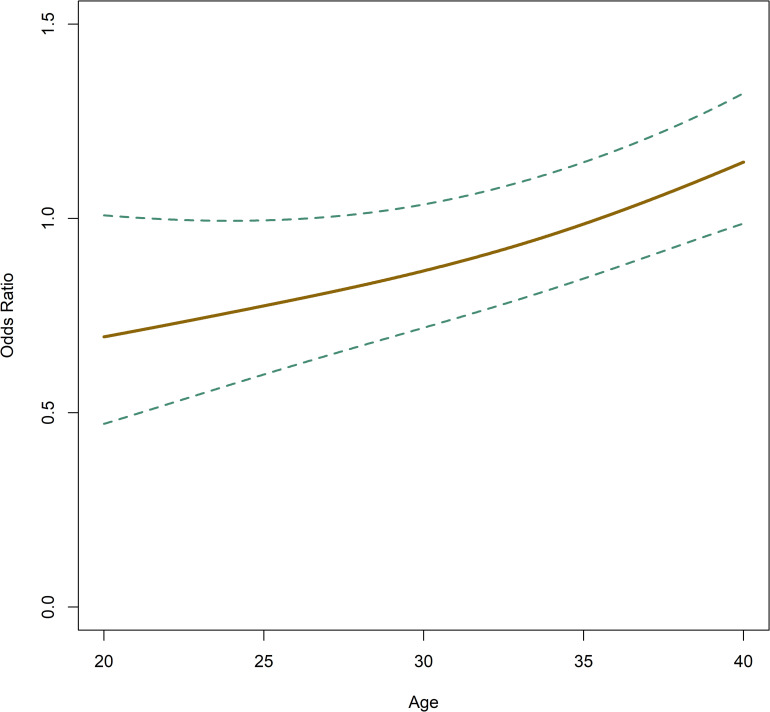
Odds ratio regarding nucleation error occurrence relative to patient age. Dashed lines show the 95% confidence intervals (CI).

In accordance with Table 4, Fig 5 shows a distinct decline in LB ratio with age and Fig 6 shows an increased incidence of NE with age.

Both the use of surgically retrieved spermatozoa (PESA, TESA or TESE) and the use of oocyte insemination (ICSI) was associated with a higher incidence of NE than ejaculated spermatozoa, with multivariate OR values of respectively 2.41 (*P* < 0.0001) and 1.80 (*P* < 0.0001), see Table 2. This is further elaborated in Table 5, showing that ICSI and surgical retrieving of sperm leads to a higher NE occurrence. Additionally, Table 5 illustrates that the average t2 timing is correlated (*P* < 0.002, from Table 2) with NE occurrence. Table 5 further indicates a connection between NE and increasing occurrence of short and long second cell cycles, which is not statistically significant, though (Table 2).

**Table 5:** Association between insemination type and live birth, nucleation error occurrence, short second cell cycle (t3—t2 < 9.33 h) and long second cell cycle (t3—t2 > 12.65).

Outcome	Insemination type	N	LB rate (%)	NE (%)	Average t2 (h)	Short cycle (%)	Long cycle (%)
LB	IVF	239	16.9	12.1	26.9	1.2	11.3
	ICSI*	259	19.1	17.0	26.3	5.0	15.4
Not LB	IVF	1174	-	20.2	28.1	8.2	22.7
	ICSI*	1097	-	31.5	27.9	12.5	24.6

*Including PESA, TESA and TESE.

No statistically significant association was seen between BMI and NE occurrence (Table 2). The LB rate however declines with increasing BMI (OR = 0.98, *P* < 0.05).

**Fig 7:**
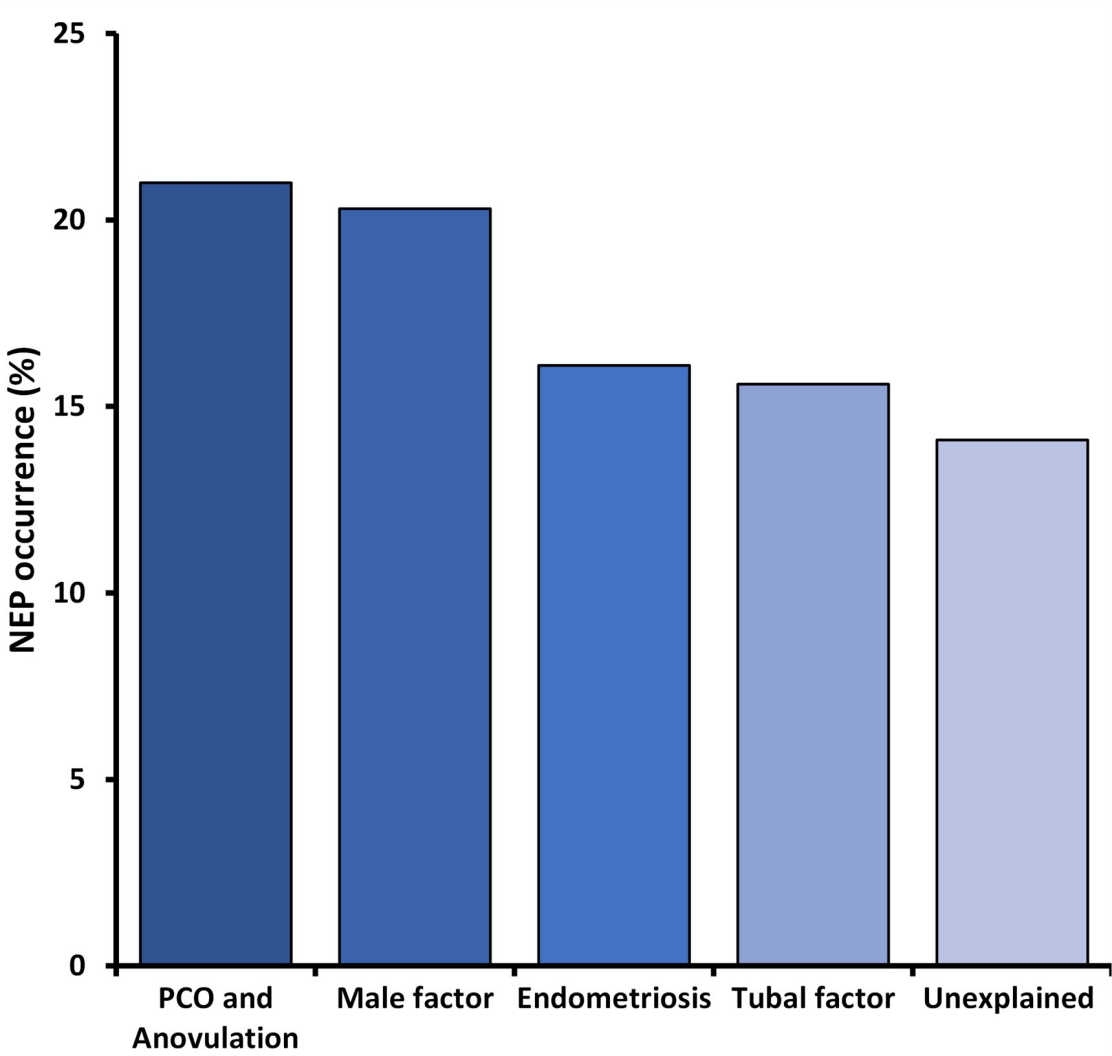
Nucleation error phenotype (NE) occurrence at infertility diagnoses.

The various etiologies of infertility (Fig 7) were not significantly distinctive regarding LB.

There are no statistically significant differences in NEP occurrences for the different etiologies of infertility. Further, no univariate statistically significant association could be seen between type of ovarian stimulation protocol (agonist or antagonist) and NE occurrence (23.4% vs. 24.0%; *P* = 0.5).

Day 2 transfers have lower average LB rates than later transfers (OR = 0.69, *P* < 0.003; Table 1). The NE occurrence is lower for day 2 transfers (OR = 0.74, *P* < 0.006; Table 2).

#### Blastomere asymmetry

Uneven sized blastomeres at the 2-cell stage yields an OR for live birth of 0.55 (univariate) respectively 0.76 (multivariate). The associated *P* values are < 0.001 resp. 0.24. Hence, in this case the distinct statistical significance from a univariate analysis asymmetry is absent when considering several variables (covariates).

When considering NE predictions for uneven sized blastomeres at the 2-cell stage (S3 Table), the OR is 1.62 (univariate) respectively 1.58 (multivariate). The associated *P* values are < 0.001 respectively > 0.003.

**Fig 8:**
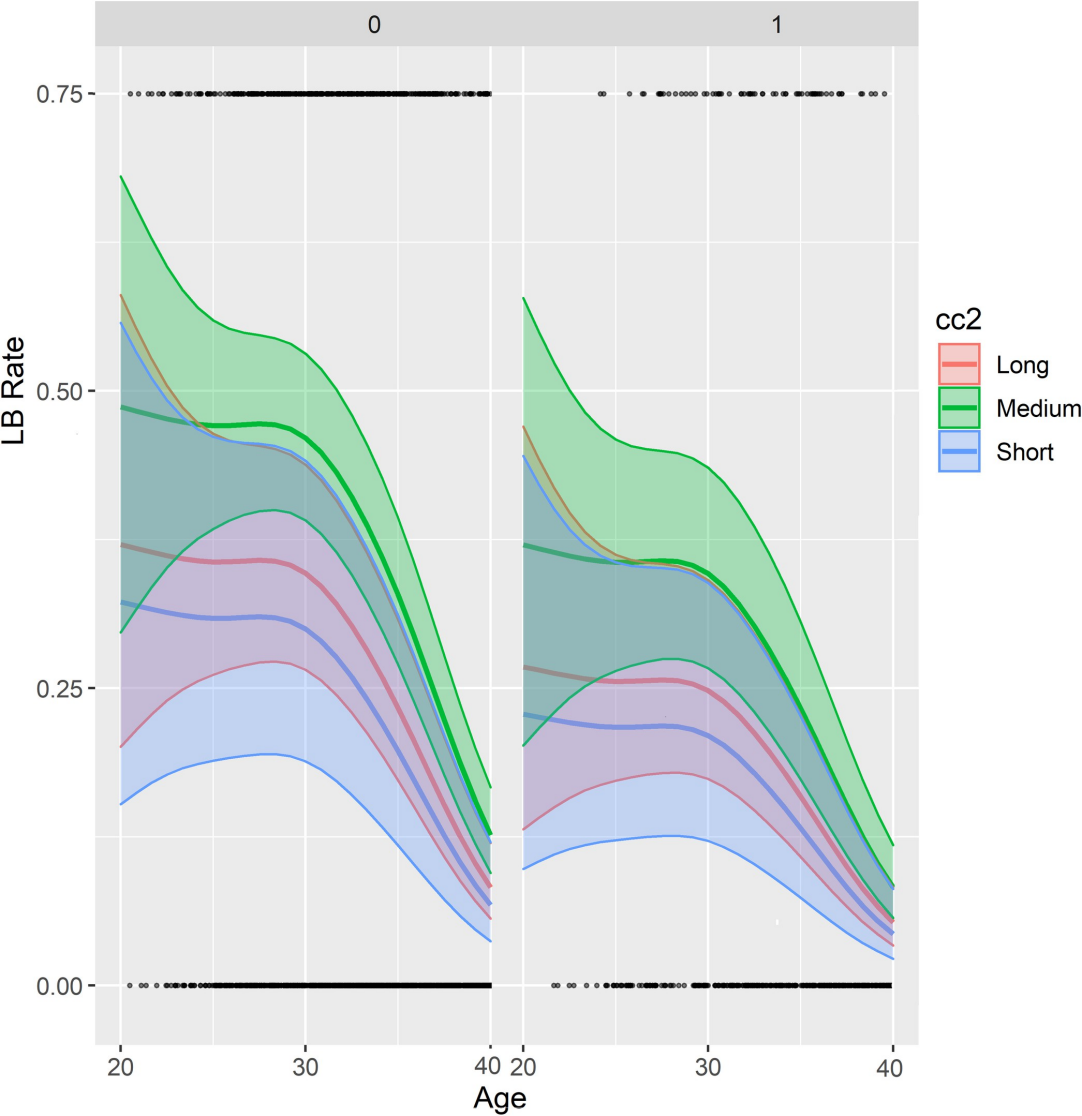
GAMM model fit regarding live birth relative to age, second cell cycle (cc2) duration (long, medium, short) and nucleation error. The nuclear errors occur on the right side of the figure. The 95% confidence interval (CI) are outlined in red, green, and blue.

Fig 8 illustrates aggregated dimensions of a GAMM model fit regarding live birth relative to age, second cell cycle (cc2) duration (long, medium, short) and nucleation error.

## Discussion

This retrospective study assessed the incidence of NE and NEPs observed by TLI and their association with LB-KID rates. We found that embryos exhibiting NE at the two-cell stage on average had distinctly lower LB-KID rates compared to embryos with no evident NE. In addition, maternal age, oocyte insemination using ICSI or surgically retrieved spermatozoa, late cleavage (t2) recordings and uneven sized blastomeres at the two-cell stage showed an increased incidence of NE. Transfers at Day 2 exhibited a decrease in NE occurrence, relative to later transfers.

The observed frequency of NE among transferred embryos in our study (23.8%) is lower than recorded in previous studies [18, 24]. Interestingly, both these studies predominantly comprised oocyte donation cycles and/ or cycles utilising only intra cytoplasmic sperm injection (ICSI). In our study, on the contrary, data from all treated couples frequenting our IVF clinic were included, presumably representing a broader infertile population with varying ages and etiologies. Additionally, the laboratory environment e.g., culture media, gas concentrations, ICSI procedure etc. can vary between IVF laboratories and these factors may influence the frequency of NE error occurrence among clinics [24].

In our analysis, live birth was the primary outcome. Our retrospective analysis share some features with the study of Aguilar et al. [18], although implantation rates were used as their main outcome measure. Aguilar and colleagues found no association between two-cell multinucleation and embryo implantation rate, contradictory to our results on live birth. However, the significant reduction in LB-KID associated with nucleation errors in our analysis is in concordance with both Ergin et al. [23] and Desch et al. [24]. Patient age and presence of multinucleation were the only significant predictors of clinical pregnancy [23]. A multivariate analysis performed by Desch et al. [24] also suggested that women’s age, time of appearance of two-cells and multinucleation status at the four-cell stage were key determinants of embryo’s live birth potential.

This study predominantly comprised Day 2 embryo transfers and thereby allowed evaluating the importance of NE in early pre-implantation embryos. Fauque et al. [25] also reported embryo transfer of early cleaved four-cell embryos on Day 2, suggesting that embryos with minimal fragmentation, but with NE had a lower live birth probability than those without NE [25]. These findings align with ours, underlining the significance of including NE evaluations alongside morphological attributes for early pre-implantation embryo selection.

The 31% lower probability of LB for Day 2 embryo transfers as evidenced in our study may be improved by prolonged culture until Day 5 blastocyst stage. There is limited information regarding use of Day 2 and 3 transfers relative to blastocyst transfers, but according to The European IVF-monitoring Consortium (EIM) for Assisted reproductive technology in Europe for 2016 [46] only 41.9% comprised of fresh blastocyst transfers.

Fertility clinics in the United States have preferentially transitioned to transferring blastocysts over cleavage stage embryos, cleavage stage embryo transfers are still performed in various parts of the world [47]. This could indicate a higher prevalence of Day 2 and Day 3 transfers for Europe, relative to the United States.

There is probably also a general shift in the practice of day of embryo transfer from early to late stages of embryo development, primarily due to higher implantation and live birth rates, as well as reduction in multiple pregnancy rates due to improved embryo selection, restriction of number of embryos transferred as well as better synchronicity between the stage of transferred embryo and endometrial receptivity [48]. The ultimate aim of IVF treatment is the birth of a healthy singleton and not just an embryo transfer, and hence translating our findings to aid blastocyst selection and transfer might be beneficial for the patient.

However, whether prolonged culture until blastocyst stage is suitable and justifiable in all treatment cycles, even those with low number of oocytes retrieved and sub-optimal embryo quality is debatable [49]. In spite of the benefits given to extended culture, the primary clinical disadvantage of the rationale behind offering only blastocyst transfers is the potential cancellation of an embryo transfer that would have resulted in a live birth [50]. The choice of cleavage stage or blastocyst embryo transfer for poor prognosis patients becomes even more arduous with increased likelihood of cycle cancellations, financial burden as well as emotional and psychological toll on patients [47].

It has been suggested by Aguilar et al. [18] that two-cell embryos have an efficient error restoration system compared to later cell stages. We found an 89.6% reversibility of NE at the two-cell stage. They observed a reversibility of NE in 73.4% of the embryos, suggesting a possible reparation process [18]. This is accentuated by the 73 live births reported in our study from embryos exhibiting NE. Additionally, the mechanistically distinct modes of origin of binucleation at the 2-cell stage or at later stages of pre-implantation embryo development could explain the disparity in their embryo development [51]. Presence of binucleation in mid- to late- preimplantation developmental stages indicate chromosomal segregation errors and subsequent aneuploidy in blastocyst stages, whereas binucleation at the 2-cell stage due to cytokinesis failure may have minimal impact on developmental potential [51]. The presence of extra- nuclear DNA observed infrequently in normally developing blastocysts, intermediately evident in good quality cleavage stage embryos and highly frequent in arrested embryos suggest the advantage of real time imaging of the nucleation status of embryos combined with analysis of cell types at the blastocyst stage [52].

The decision making for embryo selection during the study period was based on clinician’s preferences and the flexibility in scheduling transfer day at the convenience of both the patient and the clinician. An increased practice of performing frozen embryo replacements in the United States, accounting for approximately 70% of ART cycles reveal a shift in treatment practices [53]. Decision making also based on embryo development progression until the blastocyst stage should be the ultimate goal in any clinical practice and this will aid clinicians in patient counselling [54] as well as achieving better clinical outcomes. Additionally, carrying out robust prospective randomized trials and the inclusion of cumulative live birth data will make the study findings more clinically applicable.

The concept of self-correction has also been supported by FISH (fluorescence in situ hybridisation) studies, suggesting the restoration of ploidy during early pre-implantation development [55, 56]. The transient process of NE reversibility might also involve rearrangement of chromosomal anomalies [57–59] leading to mononucleated blastomeres at the four-cell stage [60, 61] and subsequent successful deliveries [57, 58, 60]. The nucleation errors present in the 2-cell stage, even if corrected in the 4-cell embryo, maybe evident in the quality of blastocysts. The higher incidence of 2-cell multinucleation and a slightly reduced incidence of the nucleation error during the transition to 4- cell stage and the possible self-correction mechanism provides a more significant prediction of aneuploidy in the embryo at 4- cell stage [62]. This is further strengthened by the conclusion drawn by Tvrdonova and colleagues [62] that the incidence of nucleation error in the group of euploid embryos that resulted in a clinical pregnancy in the four-cell stage is 7 times lower than in the group of aneuploid embryos. Hence, additional emphasis must be given to this concept in large scales studies aimed at later embryonic stages.

Our analysis of different NE types and their relationship with LB-KID did not reveal statistically significant associations. This could change with a larger dataset. The type of nucleation error phenotype (NEP) reported in earlier studies was hypothesised to originate from the disruption of intracellular restructuring, remodelling, and imprinting in the developing oocyte [14]. The possible repair mechanism reported in previous studies was hypothesised to lead to resumption of cytokinesis, thereby increasing the probability of having a genetically normal blastomere when only one of the blastomeres is affected [63].

The association between NE occurrence and the timing of morphokinetic variables in our analysis is in line with previous published studies [18, 23]. Statistically significant differences in t2, t4 and t6 between embryos exhibiting NE and those not exhibiting NE were reported by Ergin et al. [23]. These findings were subsequently confirmed by Aguilar et al. [18]. When NE evaluation was performed between 25 and 27 hours post insemination based on ESHRE/ALPHA consensus embryo check time limits, only 27.6% of the embryos exhibiting NE were detected [23]. This means that there was a non-detection of 72.4% of the NEs in transferred embryos, due to a median onset of NE occurrence of 30.6 hours and a median end of NE occurrence was 36.2 hours, which is a considerably later interval than the consensus check time. As a related finding, our analysis showed a distinct increase in NE occurrence when t2 was delayed (≥ 29.42 hpi).

Additionally, the impact of normal sperm, impaired ejaculated sperms as well as testicular sperms on early embryo morphokinetics must be considered, especially when the study population is heterogenous, with male and female infertility aetiologies, including azoospermic males where testicular sperms were used to attain fertilization. Differences in morphokinetic parameters on the embryonic cell cycle [64, 65], higher aneuploidy rates and mosaicism reported in cases with low sperm concentrations [66] highlights the need for incorporating sperm origin while developing novel morphokinetic models for embryo selection.

Additionally, our retrospective analysis reveals the significance of two-cell NE on live birth rates. However, the embryo selection algorithm, published by Meseguer et al. [36], only includes four-cell multinucleation as a deselection criterion for embryo selection. These findings along with our study findings point to the importance of including NE at the two-cell stage along with NE at the four-cell stage in the design of morphokinetic algorithms for embryo selection. Based on our study conclusion, we second the proposal by Ergin et al. [23] to include two-cell NE as a parameter for TLI based embryo grading schemes.

In our analysis, a lower proportion of embryos exhibited NE at the four-cell stage, compared with NE at the two-cell stage. This is probably due to embryo selection bias, with embryologists selecting embryos with no NE over those with NE. There might also occur an additional selection bias by embryologists who, based on ESHRE/ALPHA consensus morphology grading (2011) [67] scheme, choose embryos with lesser fragmentation and / or symmetrical blastomere sizes at the two- cell stage.

Our study demonstrated that an uneven symmetry of the blastomeres at the two-cell stage is associated with a higher incidence of NE. This strengthens the findings from earlier studies that unevenly sized blastomeres have a positive correlation with higher frequencies of multinuclearity [68, 69]. Interestingly, there is no statistically significant association between blastomere symmetry and LB.

The seemingly clear (*P* < 0.001) decrease in live birth rates observed with uneven sized blastomeres at the 2-cell stage in the univariate analysis turned out to be non-significant when applying a multivariate approach. This exemplifies the limitations of univariate analyses. In general, a seemingly strong association can be caused by other covariates, therefore the univariate results should be interpreted with reservations.

Our analysis further emphasizes the significance of including morphological embryo selection criteria described by ESHRE/ALPHA consensus grading (2011) as well as information gathered from previously published studies [28, 69] in early preimplantation embryo selection schemes.

Additionally, we found no differences in the occurrence of NE, relative to ovarian stimulation regimes used. This is in accordance with findings by Kyrou et al. [70] and Sun et al. [71]. However, our finding regarding ovarian stimulation contrasts the hypothesis forwarded by De Cassia et al. [63] that the development of NE may be due to specific IVF cycle parameters, leading them to claim an impairment in follicle recruitment and selection in agonist cycles compared to a less discriminating selection in antagonist cycles.

Considering the effect of maternal age on NE occurrence, we found an increased incidence of NE and a substantial reduction in LB-KID, when using age as a variable in the multivariate analyses. In accordance with our findings, Balakier and colleagues reported that the frequency of NE occurrence was more pronounced in women ≥ 40 years of age than in women ≤ 35 years of age [60]. The importance of women’s age as a critical factor in live birth was further confirmed by Fauque et al. [25], reporting no births observed when multinucleated embryos were transferred on Day 2 for women ≥ 35 years old. However, our findings contrast with previously published studies that reported no impact of female age on embryo multinucleation [14, 23, 28].

Surgically retrieved sperm samples (TESA) have been presumed to result in higher occurrence of NE, possibly due to retrieval of immature spermatozoa with centrosome defects [63]. In our analysis, inseminating oocytes with ICSI or surgically retrieved spermatozoa was associated with a profoundly higher incidence of NE relative to oocytes that were inseminated with ejaculated spermatozoa. This contradicts conclusions drawn by both De Cassia et al. [63] and Fauque et al. [25] that sperm origin was not a statistically significant factor determining an embryo’s fate. Interestingly, there is no statistically significant difference in live birth rate from the multivariate analyses between different origins of spermatozoa (ejaculation with or without ICSI and surgically retrieved spermatozoa).

Our analysis underlines the effect of selection bias based on clinical practice. This is evident for any retrospective study of this kind, making the comparison between such studies complex. We also emphasise the importance of considering the high degree of observed variation in embryo development due to patient dependent factors, especially for retrospective time-lapse studies as suggested by Kirkegaard et al. [72]. Treatment related factors and culture conditions have also been associated with variations in division timings studies [73–75].

In addition, the exclusion of partial implantation cycles in this KID analysis poses its own issues. For example, double embryo transfers (DET) where the transfer of an embryo with nucleation error alongside another embryo with no visible NE resulting in only one live birth could not be included in the KID analysis. The exclusion of these partial implantations leads to over-representation of non-implantations [43], see Sayed et al. [31] for an elaboration of the pros and cons of using KID data.

Additionally, the effect of NE on perinatal outcomes should be assessed. Seikkula et al. [76] reported comparable ongoing pregnancy rates from binucleated, multinucleated and mononucleated embryos [76]. Further culturing of these two-cell embryos exhibiting NE to the blastocyst stage for either fresh embryo transfers or vitrifying them for future frozen embryo transfers would help establish their live-birth and perinatal outcomes. This becomes imperative as aneuploidy testing is restricted in many countries, leading to dependence on non-invasive technologies and tailored embryo selection criteria to augment clinical outcomes.

In conclusion, our retrospective analysis revealed a significant association between NE of Day 2 embryos and live birth following IVF treatment. We found several factors associated with NE occurrence and LB-KID rates. These factors may be included in strategies used for embryo selection and cryopreservation. A better understanding of the dynamic process of nuclear formation and their continuous monitoring using Time-lapse imaging technology may provide better criteria for embryo selection, especially where genetic screening is not available for the general population of patients seeking fertility care.

## Supporting information

S1 Table Association of second cell cycle duration (cc2) derived from EEVA model with nucleation error (NE) occurrence and live birth (LB) rates. (DOCX)

S2 Table Association between degree of embryo fragmentation, nucleation error (NE) occurrence and live birth (LB) for day 2 embryos. (DOCX)

S3 Table Association between blastomere symmetry (even/uneven) at t2 (appearance of two cells) and nucleation error (NE) occurrence. (DOCX)
