# Supplementary material for: Nucleation status of Day 2 pre-implantation embryos, acquired by time-lapse imaging during IVF, is associated with live birth
Source: PLoS One. 2022 Sep 22;17(9):e0274502. doi: 10.1371/journal.pone.0274502 (PMC9498959; doi:10.1371/journal.pone.0274502)
Supplement: S1 Table — (DOCX) [file pone.0274502.s001.docx]

**S1 Table. Association of second cell cycle duration (cc2) derived from EEVA model with nucleation error (NE) occurrence and live birth (LB) rates.**

| **Classifier cc2**  **(Hours)** | **Number of embryos** | **Nucleation errors (NE) %** | **LB rate**  **(%)** |
| --- | --- | --- | --- |
| cc2 <9.33 hours  EEVA model short cycle | 195 | 29.7 | 8.2 |
| cc2 >= 9.33 hours  EEVA model short cycle | 2412 | 22.8 | 19.6 |

*P* < 0.001 for LB; *P* < 0.05 for NE

**EEVA: Early Embryo Viability Assessment** (cc2<9.33h; VerMilyea *et al.,* 2014).
