# Supplementary material for: Nucleation status of Day 2 pre-implantation embryos, acquired by time-lapse imaging during IVF, is associated with live birth
Source: PLoS One. 2022 Sep 22;17(9):e0274502. doi: 10.1371/journal.pone.0274502 (PMC9498959; doi:10.1371/journal.pone.0274502)
Supplement: S2 Table — (DOCX) [file pone.0274502.s002.docx]

**S2 Table. Association between degree of embryo fragmentation, nucleation error (NE) occurrence and live birth (LB) for day 2 embryos.**

| **Degree**  **of fragmentation**  **(%)** | **Number of embryos** | **Nucleation error (NE) %** | **LB rate**  **(%)** |
| --- | --- | --- | --- |
| 0-10% | 1321 | 22.3^c^ | 20.7^a^ |
| 10-20% | 999 | 23.2^c,d^ | 17.3^a,b^ |
| >20 % | 445 | 28.8^d^ | 11.7^b^ |

a: *P* < 0.05

b: *P* < 0.01

c: NS

d: *P* < 0.05
