# Supplementary material for: Nucleation status of Day 2 pre-implantation embryos, acquired by time-lapse imaging during IVF, is associated with live birth
Source: PLoS One. 2022 Sep 22;17(9):e0274502. doi: 10.1371/journal.pone.0274502 (PMC9498959; doi:10.1371/journal.pone.0274502)
Supplement: S3 Table — (DOCX) [file pone.0274502.s003.docx]

**S3 Table. Association between blastomere symmetry (even/uneven) at t2 (appearance of two cells) and nucleation error (NE) occurrence.**

| **Blastomere symmetry** | **Number of embryos with no visible NE** | **Number of embryos with visible NE** | **Nucleation error**  **(NE) %** |
| --- | --- | --- | --- |
| Even blastomere size at t2 | 2507 | 561 | 22.4 |
| Uneven blastomere size at t2 | 262 | 95 | 36.3 |

*P* < 0.001; Odds Ratio = 1.62
